# Case report: Successful outcome of COVID-19 in the context of autologous hematopoietic stem cell transplantation: The impact of the anti-SARS-CoV-2 vaccine and early remdesivir

**DOI:** 10.3389/fmed.2022.944855

**Published:** 2022-07-22

**Authors:** Angioletta Lasagna, Antonio Piralla, Simona Secondino, Paolo Sacchi, Fausto Baldanti, Raffaele Bruno, Paolo Pedrazzoli

**Affiliations:** ^1^Medical Oncology Unit, Fondazione IRCCS Policlinico San Matteo, Pavia, Italy; ^2^Microbiology and Virology Department, Fondazione IRCCS Policlinico San Matteo, Pavia, Italy; ^3^Division of Infectious Diseases I, Fondazione IRCCS Policlinico San Matteo, Pavia, Italy; ^4^Department of Clinical, Surgical, Diagnostic and Pediatric Sciences, University of Pavia, Pavia, Italy; ^5^Department of Internal Medicine and Medical Therapy, University of Pavia, Pavia, Italy

**Keywords:** hematopoietic stem cell transplantation (HSCT), remdesivir, G-CSF, neutropenia, COVID-19, vaccine

## Abstract

Coronavirus disease (COVID-19) in patients undergoing hematopoietic stem cell transplantation (HSCT) is a major issue. None of the published papers have reported data on the outcome of HSCT patients with COVID-19 according to the vaccination status and the short course of remdesivir (RDV). Therefore, we present the case of a 22-year-old man with relapsed testicular non-seminomatous germ-cell tumor who was diagnosed with COVID-19 during his first auto-HSCT. Our case report is the first one describing the efficacy of early RDV (and its anti-inflammatory effects that might counterbalance the negative effect of the recombinant human granulocyte-colony stimulating factors -rhG-CSF-) in the context of severe neutropenia following HSCT with the concomitant onset of COVID-19.

## Introduction

Coronavirus disease (COVID-19) in patients undergoing hematopoietic stem cell transplantation (HSCT) is a major issue. A systematic review has reported that the mortality rate of COVID-19 was 17% (95% CI = 0.12–0.24) in auto-HSCT and 21% (95% CI 0.16–0.25) in allo-HSCT recipients, with a median time from HSCT to SARS-CoV-2 infection ranging from 16 to 23 months (1). One of the largest observational cohort studies reported by the Center for International Blood and Marrow Transplant Research (CIBMTR) summarized the clinical outcome of HSCT recipients with COVID-19 from six countries, confirming the higher mortality among HSCT recipients compared with the general population (2). Since the outbreak of the COVID-19 pandemic, the Infectious Diseases Working Party (IDWP) of the European Society for Blood and Marrow Transplantation (EBMT) drafted guidelines to support the management of transplant candidates and recipients (3). At the time of the publication of the EBMT recommendations, the authors advocated close collaboration with specialists in infectious diseases to manage the therapy in transplanted patients with concomitant COVID-19 infection. In particular, despite the Food and Drug Administration (FDA) approval of remdesivir (RDV), there were doubts about the role of RDV and the treatment schedule in this particular set of patients (3). Nowadays, the possibility to prevent the unfavorable evolution of COVID-19 in high -risk patients is based on the early administration of monoclonal antibodies and antiviral drugs (4). To date, only one case of SARS-CoV-2 infection has been described with a favorable outcome during conditioning chemotherapy before HSCT in solid tumors (5). In this case report, the positivity for SARS-CoV-2 was detected during the conditioning chemotherapy (day 2 of 3) and the infectious disease consult recommended the use of RDV according to the final report published in 2020 (6), but with the 3-day course as suggested by the PINETREE trial (7). None of the published papers have reported data on the outcome of HSCT patients with COVID-19 according to the vaccination status and the short course of RDV.

## Case description

Therefore, we present the case of a 22-year-old man with relapsed testicular non-seminomatous germ-cell tumor who was diagnosed with COVID-19 during his first auto-HSCT.

He was admitted to our Medical Oncology Unit on 3 January 2022, 1 month after the second dose of the BNT162b2 anti-SARS-CoV-2 vaccine. Upon admission, the nasopharyngeal swab was negative for SARS-CoV-2 using the real-time RT-PCR (SARS-CoV-2 Elite MGB kit, ELITechGroup Molecular Diagnostics) and the SARS-CoV-2 IgG-specific antibody level was 860 BAU/ml (negative if < 0.79 BAU/ml). The patient did not have other risk factors for severe COVID-19. On 16 June 2021, the patient underwent right orchifunicolectomy surgery for a mixed germline tumor. After the surgery and complete remission of tumor markers, the patient was treated with systemic therapy based on bleomycin, etoposide, and cisplatin (PEB). On 24 November 2021, a CT scan revealed bone metastases, and autologous HSC transplantation was considered. The mobilization consisting of two cycles of chemotherapy with paclitaxel and ifosfamide was used with success. On 6 January 2022, the myeloablative conditioning chemotherapy, consisting of carboplatin AUC18 and etoposide 1,350 mg/m^2^, was started. On “day 0,” a total of 4.6 × 10^6^/kg bodyweight CD34+ cells were infused, and the day after, the patient received a daily subcutaneous filgrastim (granulocyte colony-stimulating factor; G-CSF). He also started the antibiotic prophylaxis with fluoroquinolone (levofloxacine 500 mg/die) and the antifungal prophylaxis with fluconazole (200 mg/die) to reduce the risk of bacterial and invasive fungal infections (IFIs) according to the guidelines (8, 9).

**Figure 1:**
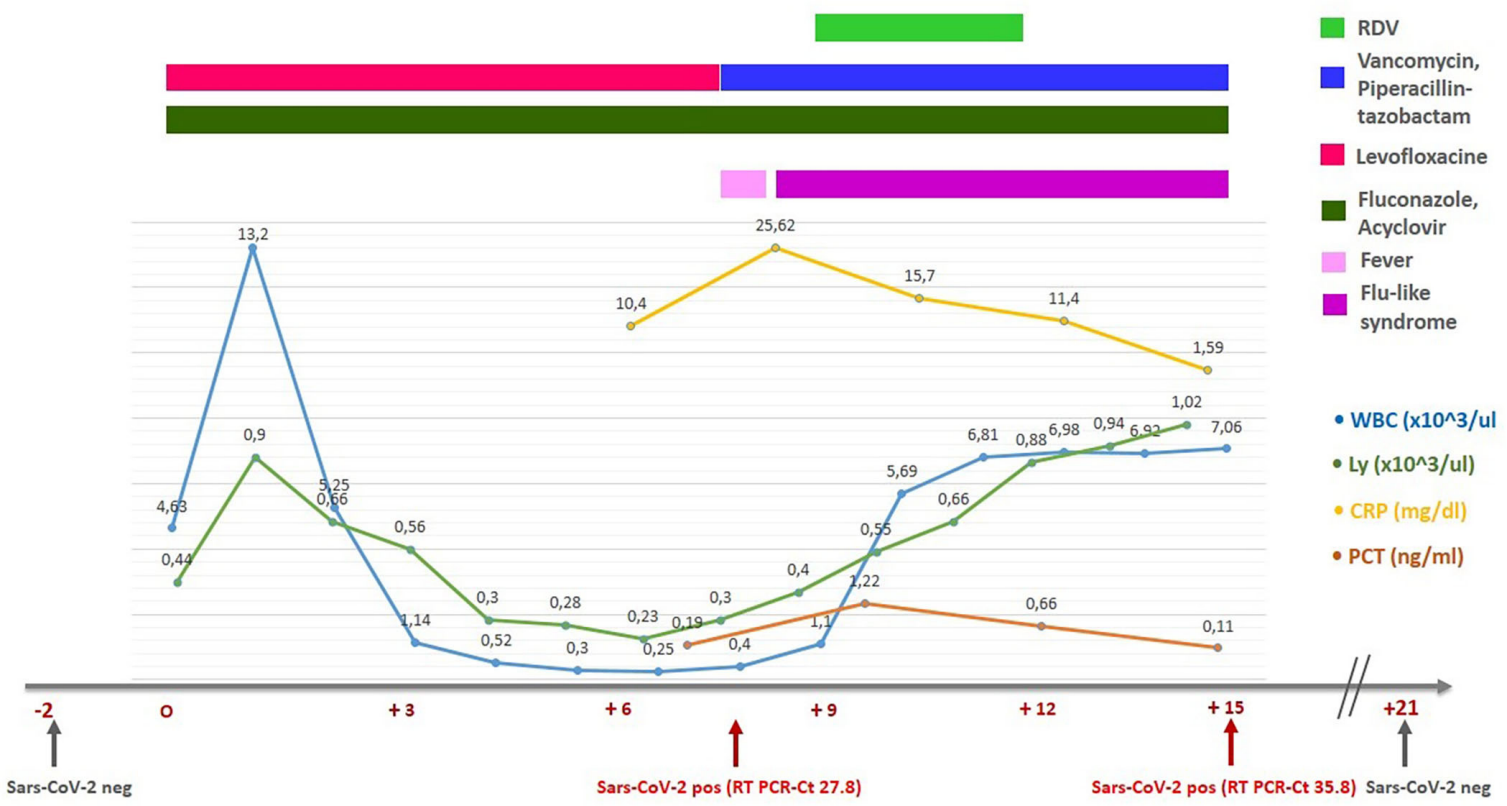
Clinical outcome in patient during SARS-CoV-2 infection.

On “day +8,” during the neutropenic phase, he developed a fever (38.0°C). Antibiotic therapy with vancomycin and piperacillin/tazobactam was initiated. Blood and urine cultures and beta-D-glucan (BDG) were all negative, while his swab became positive for SARS-CoV-2 with a cycle threshold (Ct) value of 27.8. The presence of the SARS-CoV-2 Omicron variant (B.1.1.529) was confirmed by genomic sequencing. The chest radiography did not demonstrate SARS-CoV-2-specific alterations and oxygen saturation remained stable at 98–99%. As regards the cytokines panel, in our unit, we could only dose interleukin-6 (IL-6). At the time of the detection of the COVID-19 infection, IL-6 was 18.22 [0.00–3.12] pg/ml. As prescribed by the infectious disease consultant, the patient received intravenous RDV (200 mg on the first day and 100 mg on the second and the third days), as suggested by the recently published PINETREE trial for the early treatment of patients at high risk (7). He reported mild flu-like symptoms (rhinorrhea and sore throat) and his swab on “day +15” demonstrated an RT-PCR Ct value of 35.8. No transaminase elevation was observed. On “day +16,” he was discharged in asymptomatic clinical conditions with a decreased value of IL-6 (2.88 pg/ml). The main laboratory data during the first auto-HSCT and the timeline are summarized in Figure 1. The patient repeated a swab 14 days after the COVID-19 negative for SARS-CoV-2.

Written informed consent was obtained from the patient for the publication of this case report and any accompanying laboratory findings.

To date, the patient has completed his third auto-HSCT, and the patient is alive without progression. The laboratory examination does not reveal evident abnormalities. Next, we consider the post-transplantation maintenance therapy with etoposide, hoping to achieve the complete remission of the tumor. Before the second autotransplant procedure, SARS-CoV-2-specific IgG antibody level was significantly higher than that observed before the COVID-19 infection (1,960 BAU/ml), probably due to the “hybrid immunity.” A slow decline was observed before the third autotransplant procedure (IgG levels of 980 BAU/ml). At the time of the submission of this article, the patient has not received the third dose yet, because, according to the guidelines of the Italian Ministry of Health, at least 4 months must pass from the infection (10).

## Discussion

To the best of our knowledge, this is the first case report of the efficacy and safety of a 3-day course of RDV in a high-risk cancer patient with COVID-19 during the early phase of autologous HSCT. As demonstrated in the PINETREE trial, a 3-day course of RDV has resulted in an 87% lower risk of death than placebo (9). In this study, the upper airway viral load was not lower in the RDV group than in the placebo group, as measured by nasopharyngeal RT-PCR testing. The time-weighted average change in viral load from baseline to day 7 was −1.24 log10 copies/ml of the respiratory sample in the RDV group and −1.14 log10 copies/ml of the respiratory sample in the placebo group (least-squares mean difference, 0.07; 95% CI −0.10 to 0.24). In our patient, the time-weighted average change in viral load from baseline to day 7 was at least 2 log10 copies (ΔCt = 8). Protracted lymphopenia leads to high viral load and prolonged shedding of replication in the immunocompromised patients (11), but in our case 14 days after the diagnosis of COVID-19 infection, the nasopharyngeal swab was negative for SARS-CoV-2. Considering the paucisymptomatic clinical conditions of our patient, we did not perform a bronchoalveolar lavage (BAL) test either at the time of the diagnosis of COVID-19 or at any later stage. Therefore, in our case, we have no data about the possible occurrence of the reversal phenomenon, defined as the possibility of having a negative nasopharyngeal swab and a positive BAL test in patients with COVID-19 (12).

Remdesivir is the monophosphoramidate prodrug of the nucleoside GS-441524, and it inhibits the viral RNA-dependent RNA polymerase (RdRp) of SARS-CoV-2 (13). The activity of RDV against the original virus (Whuan-Hu-1 prototype) and the variants of concern (VOCs) including Omicron has been demonstrated in a recent *in vitro* study (13). The role of these antiviral drugs is now becoming crucial because they retain their activity on the different SARS-CoV-2 VOCs. Indeed, this agrees with the observation that the target proteins of these antivirals are highly conserved. For the RDV key target, RdRp, there are only two amino acids changed from the ancestral lineage: P323L in all VOCs and G671S in Delta; position 4715/5063 in ORF1ab or 314/662 in ORF1b, respectively. Because they are located outside the active site of these drugs, a different susceptibility toward RDV (or also molnupiravir) is not to be expected. These results seem to suggest that the new VOCs remain sensitive to the current antiviral drugs not targeting the spike protein (13, 14). The development of new pan-corona antivirals will become a complementary strategy to the vaccine and monoclonal antibodies to protect frail patients and to control the ongoing pandemic.

The favorable clinical course of our patient has confirmed the efficacy of this antiviral against VOCs *in vivo*.

Vaccination against SARS-CoV-2 is currently one of the best weapons against the COVID-19 pandemic. The efficacy of the COVID-19 vaccine in HSCT recipients is still debated: the humoral and cellular vaccination responses seem diminished (15). Moreover, Lindemann and colleagues demonstrated that male HSCT recipients had an impaired antibody response after two doses of the SARS-CoV-2 vaccine, while the cell-mediated response diminished both in male and female HSCT recipients (15). Our patient had received two doses of BNT162b2 anti-SARS-CoV-2 vaccine and the humoral analysis had revealed the achieved seroconversion before HSCT.

The administration of the third dose of the anti-SARS-CoV-2 vaccine to HSCT recipients might improve the immunogenicity of the vaccine, as highlighted in a recent study (16). Two doses of mRNA-based vaccines can elicit a poor neutralization of Omicron, while three mRNA vaccine doses can elicit a potent variant cross-neutralization, including Omicron (17). In our patient's case, the absence of the booster of the anti-SARS-CoV-2 vaccine might have contributed to the inadequate protection against the Omicron variant.

The role of recombinant human granulocyte-colony stimulating factors (rhG-CSF) has been widely debated since the onset of the pandemic: initially, a panel of experts had suggested the opportunity to increase the use of prophylactic rhG-CSF also in the case of chemotherapeutic regimens with <20% chance of inducing febrile neutropenia to minimize neutropenia duration (18). At the same time, however, some authors began to speculate on the effect of rhG-CSF on the neutrophil count and the relationship between the rising neutrophil-to-lymphocyte ratio (NLR), the excess of neutrophil extracellular traps, and the worst clinical outcome during the COVID-19 infection (19). Some studies described the possibility of worsening of the clinical conditions in cancer patients with concomitant COVID-19 after the use of rhG-CSF due to an imbalance in the inflammatory response (20–22). Zhang and colleagues reported that the administration of rhG-CSF among hospitalized patients was linked to an increased risk of death (HR: 3.56, 95% CI: 1.19–10.2, *p*-value: 0.024). This effect was predominantly observed in the patients with a high neutropenia recovery due to rhG-CSF (HR: 7.78, 95% CI: 2.05–27.9, *P* value: 0.004) (21). rhG-CSF might worsen the overwhelming inflammatory reaction in COVID-19 and lead to adverse outcomes: the excessive NETosis (a type of apoptosis uniquely caused by neutrophils), induced by the epithelial and the endothelial cells affected by SARS-CoV-2, is involved in the development of the well-known “cytokine storm” (23, 24). In our case, it might be speculated that this negative effect was counterbalanced by RDV. Indeed, RDV seems to have anti-inflammatory effects against acute lung injury (ALI) *in vivo* by reducing neutrophil infiltration in the case of the Middle East respiratory syndrome coronavirus (MERS-CoV) infection (25).

## Conclusions

Our case report is the first one describing the efficacy of early RDV in the context of a severe neutropenia following HSCT with the concomitant onset of COVID-19 disease. It may be interesting for the clinicians involved not only in the management of HSCT recipients but also in the management of the neutropenia induced by the conventional chemotherapies. Moreover, this report highlights the complexity of rhG-CSF and its interaction with the immune system and the intriguing anti-inflammatory role of RDV, even in the short-course schedule.

## Data availability statement

The raw data supporting the conclusions of this article will be made available by the authors, without undue reservation.

## Ethics statement

Ethical approval was not provided for this study on human participants because Ethical review and approval was not required for the study on human participants in accordance with the local legislation and institutional requirements. The patients/participants provided their written informed consent to participate in this study.

## Author contributions

All authors listed have made a substantial, direct, and intellectual contribution to the work and approved it for publication.

## Funding

This work was partially supported by Ricerca Corrente grant no. 08067620, Fondazione IRCCS Policlinico San Matteo.

## Conflict of interest

The authors declare that the research was conducted in the absence of any commercial or financial relationships that could be construed as a potential conflict of interest.

## Publisher's note

All claims expressed in this article are solely those of the authors and do not necessarily represent those of their affiliated organizations, or those of the publisher, the editors and the reviewers. Any product that may be evaluated in this article, or claim that may be made by its manufacturer, is not guaranteed or endorsed by the publisher.
